# Evolution of Groundwater in Yinchuan Oasis at the Upper Reaches of the Yellow River after Water-Saving Transformation and Its Driving Factors

**DOI:** 10.3390/ijerph17041304

**Published:** 2020-02-18

**Authors:** Lina Mi, Juncang Tian, Jianning Si, Yuchun Chen, Yinghai Li, Xinhe Wang

**Affiliations:** 1 School of Civil and Hydraulic Engineering, Ningxia University, Yinchuan 750021, China; xiaomizi-123@163.com; 2 Efficient Uses of Water Resources in Arid Modern Agriculture Ministry of Educational, Engineering Research Center, Yinchuan 750021, China; 3 Groundwater Monitoring Department, Hydrological and Water Resources Monitoring and early Warning Center of Ningxia, Yinchuan 750001, China; sijianning@126.com (J.S.); nxchenyuchun@126.com (Y.C.); 4 Comprehensive Study Office, Ningxia Hydro-environmental Geological Survey Institute, Yinchuan 750021, China; wangxinhe9511@163.com

**Keywords:** Yinchuan Oasis, agricultural water-saving, groundwater, groundwater storage, ecological environment

## Abstract

In recent years, the amount of water diverted from the Yellow River has been decreasing year by year, which is the biggest problem for the development and utilization of water resources in Yinchuan Oasis (YCO). Through the implementation of the Agricultural Water-saving Transformation Project (AWSTP), water resource shortage in the YCO has been alleviated greatly, and ecological degradation problems, such as soil salinization, have also been effectively addressed. However, how the shallow groundwater in YCO has changed after the AWSTP remains unclear. This paper, based on a lot of statistical data and measured data, and by using statistical and geostatistical methods, reveals the evolution of shallow groundwater in YCO in the past 18 years (2000–2017), since the implementation of the AWSTP and its driving factors, from two aspects: groundwater dynamics and groundwater quality. The results show that compared with the initial stage of AWSTP, the amount of water diverted from the Yellow River for the YCO reduced by 36%, and accordingly, the average groundwater depth in the irrigation period increased from 0.98 m to 2.01 m, representing an increase of 1.03 m, and an average annual increase of 6cm. Moreover, the depth increase in the irrigation period is higher than that in the non-irrigation period, and that in the Northern Irrigation Area (NIA) is higher than that in the Southern Irrigation Area (SIA). Furthermore, the groundwater storage is decreasing at a rate of 855.6 × 10^4^ m^3^·a^−1^, and the cumulative storage has reduced by nearly 1.54 × 10^8^ m^3^, indicating that it is in a long-term negative equilibrium. In terms of temporal and spatial distribution of total dissolved solids (TDS) in groundwater, the TDS in SIA and NIA decreased from 1.41 g·L^−1^ and 1.84 g·L^−1^ to 1.15 g·L^−1^ and 1.77 g·L^−1^, respectively. The saline water area with a TDS above 5 g·L^−1^ and the freshwater area with a TDS below 1 g·L^−1^ decreased by 16.6 km^2^ and 334.4 km^2^, respectively, while the brackish water area with a TDS of 1~3 g·L^−1^ increased by 492 km^2^. The spatial and temporal distribution heterogeneity of TDS in groundwater is reduced and is in a slight desalinized trend overall. However, the groundwater in some areas, such as the Xingqing District, Jinfeng District, Xixia District, Yongning County, Helan County and Huinong District of Yinchuan Oasis, is at risk of further salinization. Due to the agricultural water-saving caused by the reduction of water amount diverted from the Yellow River, the groundwater recharge in YCO was reduced by 36.3%, which, together with measures such as drainage, groundwater exploitation, and industrial restructuring, drives the groundwater circulation in the YCO to a new equilibrium. This study can help us to understand the influencing process and mechanism of agricultural water-saving on groundwater systems in YCO and provide reference for efficient use and optimal allocation and management of agricultural water resources.

## 1. Introduction

Water resource plays an important role in human’s life and social development, and is the biggest challenge to ecological security and economic development [1]. Due to water resources’ shortage and overexploitation, water use conflicts between production and ecology, agriculture, and other industries, as well as the conflicts among the upper reaches, middle reaches, and lower reaches of the river basin, even in various administrative regions have become acute [2]. In countries and regions with severe water resource shortages, such problems have even evolved into war among them [3]. Some of them are suffering a series of negative ecological and environmental effects, such as ecological degradation and soil salinization. Therefore, in many water-deficient regions or developed countries, water saving has gradually been put on the agenda. Water-saving irrigation, as a method to relieve the water use conflict between humans and nature, has been popularized in arid regions around the world [4,5]. It also plays a very important strategic role in ensuring water security, food security, ecological security, and sustainable development of agriculture [6]. Various water-saving measures, such as canal lining, optimal management of canal system water delivery, improvement of irrigation plans, and adjustment of planting structure have been widely applied at home and abroad so as to improve water use efficiency [7,8], relieve the conflict between water supply and demand, expand irrigation area, promote national economic development, and improve farmlands ecological environment [9]. At present, many scholars have carried out a large amount of studies on the impact of canal lining, sprinkling irrigation, micro irrigation, and salt water irrigation on the field microclimate changes, soil moisture, solute transient in vadose zone, heat distribution characteristics, and migration laws, and have made a great deal of achievements [10,11,12,13,14,15,16,17,18,19]. While, for the impact of agricultural water-saving on groundwater, studies on groundwater levels and evaporation variation account for a relatively large proportion [20,21,22,23,24,25,26,27]. Xu and his colleagues studied the impact of water-saving on groundwater levels in Hetao Irrigation District using the lumped groundwater balance model. The results show that water-saving of the main canal system can reduce the groundwater level by 0.28–0.48 m. When water-saving technologies are adopted for both main canals and agricultural canals, the predicted groundwater level drop will be even greater, which may bring the groundwater evaporation to be reduced by 128 mm [28]. Studies by Hollanders et al. and Xu et al. suggest that the application of water-saving technologies and improvement of drainage systems can effectively reduce groundwater level and groundwater evaporation, thus better controlling waterlogging and salinization [29,30]; Xue et al. believed that water-saving irrigation in the Jiefangzha Irrigation Area in the Hetao Irrigation district of Inner Mongolia can improve regional water consumption and water productivity, while groundwater is an important source for water evaporation of shallow aquifers, accounting for more than 16% of the evapotranspiration in the irrigation area [31]. Water-related activities, such as regional-scale agricultural water-saving transformation, have caused changes in the hydrologic cycle in many irrigation areas [6,32]. In recent years, some scholars have found that, affected by agricultural water-saving, the groundwater quality and the spatial distribution of groundwater chemistry have also changed [22,33]. Li Bin, through statistical analysis, analyzed the chemical parameters of groundwater in the Hetao Irrigation Area of Inner Mongolia from 2007 to 2009 after water-saving transformation, and found that the groundwater total dissolved solids (TDS) was decreasing and the groundwater quality was desalting [34]; and Dai, by using the geostatistics method, studied the changes in groundwater levels and water quality in Jinghuiqu Irrigation Area of Shaanxi Province before and after agricultural water-saving, and believed that the groundwater level in this area was decreasing at an accelerated pace and the water quality was getting worse [6]. Groundwater plays a key role in maintaining the stability of ecosystems and human society in arid and semi-arid regions [35] and is an important water resource for maintaining local development, especially the sustainable development of local agriculture [36]. How, and to what extent, does groundwater quantity and quality in the irrigation area change after the Agricultural Water-saving Transformation Project (AWSPT) has become a new scientific problem for agricultural water conservancy in arid irrigation areas in the new era. However, there are few systematic studies on it from the perspective of regional and long temporal scales.

Yinchuan Oasis is one of the large-scale artesian irrigation areas in the inland arid region of Northwest China. More than 2000 years of Yellow River irrigation has made this region a typical artificial oasis and grain production base. Agricultural production in the irrigation area depends largely on Yellow River irrigation, and the agricultural water consumption accounts for about 93%~95% of the total amount of water diverted from the Yellow River [37]; therefore, Yellow River irrigation is fundamental to guarantee agricultural development and ecological environment health in this area. However, due to the lack of scientific and reasonable water resources management measures, the effective utilization rate of water resources is not high [38]. Long-term and large-scale diversion of Yellow River for flood irrigation, together with terrain factors, shallow groundwater depth, and unfavorable drainage, have made soil salinization the major ecological and environmental problem there since the 1960s, which has seriously affected and restricted the sustainable development of agriculture in this area [39]. The groundwater quality in this area is also deteriorating year by year, with the over-limit ratio of phreatic water TDS and hardness exceeding 50% [40]. In the end of 20th century, the National Water Allocation Policy on Yellow River was implemented [41], which regulated that the amount of water diverted to Yinchuan Oasis (YCO) should not exceed 40 × 10^8^ m^3^. After that, the amount of water diverted from the Yellow River for YCO has been greatly reduced. In order to cope with agricultural water scarcity, the Agricultural Water-saving Transformation Project (AWSTP) was implemented [42]. Improving the canal lining and drainage conditions, promoting water-saving irrigation technology, and adjusting the agricultural planting structure were the key measures. Soil salinization has been effectively alleviated after several years of AWSTP. Groundwater processes in arid and semi-arid regions are the most affected by water-saving irrigation [43]. However, there is still lack of long-term and systematic research on the groundwater quantity and quality variation from regional scale in YCO after AWSTP. Therefore, this paper intends to analyze the evolution process and driving forces of groundwater from the aspects of groundwater dynamics and quality in YCO in the past 18 years (2000–2017), so as to provide a decision-making basis for the ecological environment safety and efficient management and utilization of water resources in Yinchuan Oasis.

## 2. Research Area, Materials, and Methods

### 2.1. Research Area Overview

**Figure 1:**
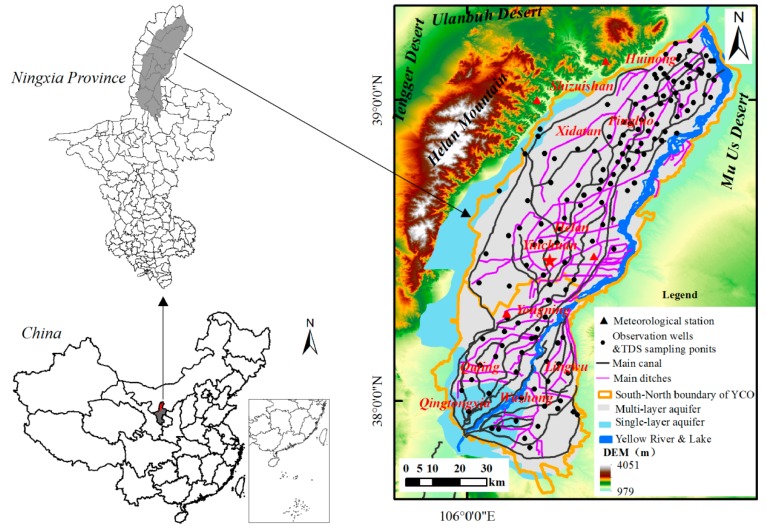
Yinchuan Oasis and the distribution of total dissolved solids (TDS) sampling points.

Yinchuan Oasis is located in the arid and semi-arid region of the upper reaches of the Yellow River, which is low in precipitation and high in evaporation, with an annual average precipitation of 180–200 mm, mostly concentrated in July to September, and a pan evaporation of 1000–1800 mm. The ecological environment there is relatively fragile. YCO is a fault subsidence basin formed during the Cainozoic era. On the west side of it is the east Helan Mountain area fault zone, on which side it is connected with the mountain massif, while the southern fault zone is located at the Northeast of Niushou Mountain, and the Northern part of it is a group of hidden faults in the south of Shizuishan. From the mountain front to the oasis, it can be divided into piedmont alluvial inclined plain, alluvial-proluvial plain, alluvial lake plain and river lake alluvial plain. It is open and flat in terrain and inclines from southwest to northeast. The entire oasis belongs to the artesian irrigation area of the Yellow River alluvial plain, with an altitude of 1090–1200 m. The Yellow River runs through the entire oasis from southwest to northeast (Figure 1). Due to the lack of surface water resources, the industrial and agricultural production in Yinchuan Oasis mainly relies on the water from the Yellow River [44]. The groundwater is mainly quaternary loose rock pore water and the thickness of quaternary loose rocks ranges from 500–700 m in front of the mountain to over 900 m in plain areas. In terms of formation lithology, the piedmont alluvial inclined plain mainly consists of pebbles, gravel, and gravel-cobbles, which are favorable in water conditions; and to the east, it mainly consists of sand gravel, gravel-cobbles, sticky sand and clay, sand clay, etc. The aquifer system is composed of phreatic water with single structure and confined water with multi-layer structures, and has a relatively complete and independent water circulation process (Figure 1). Groundwater recharge mainly comes from canals and field irrigation returned water infiltration. At present, the average depth for shallow groundwater in the irrigation period is 1.5–2.0 m, and its TDS is 1–3 g·L^−1^, and greater than 3 g·L^−1^ in local low-lying areas. Yinchuan Oasis is equipped with well-developed irrigation and drainage systems, and at present, has 6453 km of main irrigation canals. As water and salt enter the irrigation area with water diversion, in order to drain water and salt, 22 main ditches have been built, with a total length of 1176.2 km and a drainage capacity of 962.4 m^3^·s^−1^. The formation and evolution of groundwater resources in plain areas are long-term affected by the complex natural-human composite systems. As an important food production area in the Yellow River Basin, its cultivated area accounts for about 47% of the entire oasis area, with corn, winter wheat, and rice as the main food crops. Yinchuan Oasis, also known as the Qingtongxia Irrigation Area, can be divided into Northern Irrigation Area (NIA) and Southern Irrigation Area (SIA) by taking Yongning County as the boundary. The irrigation period is from April to November each year, and the non-irrigation period is from January to March and December each year.

### 2.2. Material and Method

#### 2.2.1. Data Overview

Data on water diversion and groundwater exploitation during 2000–2017 come from the 2000–2017 Water Resources Bulletin of Ningxia province [45], and data of 150 diving observation wells (monthly water table depth and annual water table variation) from 2000 to 2017. Furthermore, data on groundwater TDS in 2005 and 2017 (150 sampling points) are from the Hydrological and Water Resources Survey Bureau of Ningxia province, while statistical data on high efficiency water-saving irrigation and water productivity come from the Water Resources Departments of Ningxia province. According to the Ningxia Groundwater Bulletin [46], the groundwater recharge in Yinchuan Oasis mainly comes from canal seepage, returned water from field irrigation, and precipitation infiltration, of which, canal seepage and field irrigation returned water recharge account for about 85% to 90% of the total groundwater recharge. Therefore, we took the sum of canal seepage and field irrigation returned water recharge as the groundwater recharge in this paper. Groundwater TDS is determined by using a portable pH/conductivity multi-parameter tester (SevenGo Duo™). Water samples were collected from the 150 diving observation wells in the plain area in April of that year, the wells were basically 3–40 m deep, and the sampling was made at a depth of 10 cm below the water table (Figure 1). Then, the data on TDS could be obtained according to the conversion relationship between the conductivity and the TDS.

#### 2.2.2. Research Method

In Yinchuan Oasis, as more than 95% of water diverted from the Yellow River is used for agricultural irrigation, it was assumed in this research that the amount of water diverted from the Yellow River is the water consumed for agricultural purposes, and the reduced amount of water diverted from the Yellow River is the water saved from AWSTP.

##### Estimation of Shallow Groundwater Storage

In the estimation of shallow groundwater storage, based on the aquifer characteristics, the shallow groundwater system of YCO is taken as a whole, and it is calculated in accordance with the method of Mi et al. (2016) [47] after it being improved, namely:

(1) $$\Delta W_{i}=\overset{2}{\underset{k=1}{\sum }}\bar{\Delta H_{ik}}\cdot \mu_{k}\cdot F_{k}$$

where, Δ*W_i_* is the variation of groundwater storage in the *i*th year in Yinchuan Oasis; *k* = 1, 2, with 1 for the NIA and 2 for the SIA; *i* is the year, which is from 2000~2017; *µ* is the specific yield of phreatic aquifer in YCO, which is usually 0.039–0.051 according to the calculation results of the hydrogeological department, with 0.051 for SIA and 0.039 for NIA herein; $\bar{\Delta H_{ik}}$ is the average variation of the water table at the end of the *i*th year in SIA or NIA; *F_k_* is the area of NIA or SIA. According to the location of the observation wells, the data on groundwater level variation at the end of the year were divided into those of the NIA and SIA, respectively, and then the average obtained.

##### Geostatistics-Based Interpolation of Spatial Distribution of Groundwater Tds and Accuracy Evaluation

Geostatistics has become an important tool for hydrology, especially for large-scale hydrological studies. The Kriging interpolation is a random method based on the theory of regionalized variables, which studies the spatial structure as well as the spatial and temporal variation characteristics of observation data and their attributes with a semi-variogram model [48]. The semi-variogram is used to describe the interdependence between the observation data and their adjacent data, which is expressed as the variance of the increment spaced by *h* of the regionalized variables, denoted as γ (*h*), that is:

(2) $$\gamma \left(h\right)=\frac{1}{2N\left(h\right)}\cdot \sum_{i=1}^{N\left(h\right)}{\left[Z\left(x_{i}\right)-Z\left(x_{i}+h\right)\right]}^{2}$$

where, $Z\left(x_{i}\right)$ is the value of sampling points i; Z($x_{i}+h$) is the predicted value that is *h* (m) away from the sampling point *i*, *N* (*h*) is the logarithm of the total sampling point which is spaced by h $\mathrm{from}\;Z\left(x_{i}\right)$; and *h* is the distance between variables.

**Figure 2:**
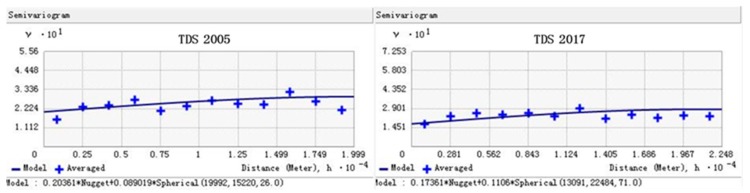
Semi-variogram fitting for groundwater TDS.

In the Arcgis geostatistics module, the Histogram analysis showed that the groundwater TDS over the two periods were normally distributed after logarithmic transformation, which meets the requirements of Ordinary Kriging interpolation. The corresponding key fitting parameters (Table 1) were obtained by fitting the semi-variogram (Figure 2) with the spherical model. The spatial correlation indicates that the spatial distributions of groundwater TDS from 2005 to 2017 are in moderate correlation, and this spatial difference is mainly caused by human activities, such as irrigation. Judging from the fitting error (Table 2), in 2005 and 2017, the mean error (ME) of TDS was basically equal to the standardized mean error (SME) and close to 0, the root-mean-square error (RMSE) is close to the average standard error (ASE) and small enough, and the root-mean-mquare standardized errors (RMSSEs) are 1.34 g·kg^−1^ and 0.91 g·kg^−1^, respectively, approximate to 1 g·kg^−1^. For more information about the calculation method of error, please refer to the Arcgis 10—Online Help Docs [49]. For large-scale research areas, this fitting error can meet the research requirements.

Via Kriging interpolation, the spatial distribution maps of TDS in 2005 and 2017 were generated and exported as raster data. Then, according to the Hydrogeology manual in China [50], and through the Arcgis statistical analysis module, the respective raster TDS map was classified into freshwater (<1 g·L^−1^), brackish water (1–3 g·L^−1^), saline water (3–10 g·L^−1^), saltwater (10–50 g·L^−1^), and brine (>50 g·L^−1^). In order to better reflect and evaluate the condition of groundwater TDS in the Ningxia province, we classified the saline water into saline water Ⅰ (3–5 g·L^−1^) and saline water Ⅱ (5–10 g·L^−1^).

##### Groundwater Recharges Estimation in the Oasis

In the Yinchuan Oasis, groundwater recharge is one of the important factors that affect groundwater dynamics. The research of Scanlon et al. suggests that in arid and semi-arid regions, precipitation recharge for groundwater is negligible if the precipitation in these regions is below 200 mm [51]. Therefore, in this research, the sum of canal seepage and return water from field irrigation is taken as the main source for groundwater recharge. The calculation method is as follows:

*W_R_* = *R_c_* + *R_f_* (3)

*R_c_* = *m·W_c_* (4)

*R_f_* = *β·Q_f_* (5)

where, *R_c_* is the recharge from canal seepage; *R_f_* is the recharge from field return water infiltration; *W_c_* is the canal water diversion; *Q_f_* is the amount of water entering the field; *m* is the recharge coefficient for canal seepage, and *β* is the recharge coefficient for field return water infiltration, and according to the calculation of the Water Conservancy Department of Ningxia province, the values of *m* and *β* are 0.20 and 0.26, respectively.

## 3. Results analysis

### 3.1. Dynamics of Shallow Groundwater in Yinchuan Oasis after AWSAT

#### 3.1.1. Characteristics of the Temporal and Spatial Variation of Shallow Groundwater Depth

**Figure 3:**
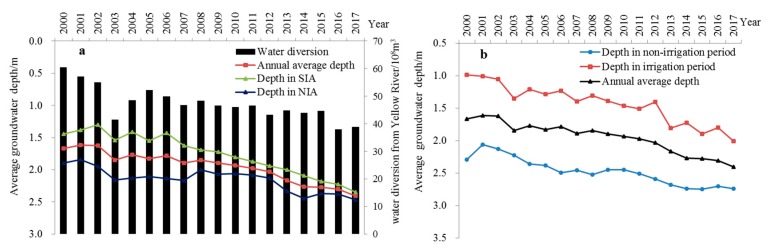
Annual average variation of groundwater depth during 2000–2017 in Yinchuan Oasis: (**a**) Groundwater depth variation between Southern and Northern Irrigation Areas, (**b**) Groundwater depth variation during irrigation and non-irrigation period.

The variation of groundwater levels is a direct reflection of the variation of groundwater dynamics. Figure 3a shows that compared with 2000, the amount of water diverted from the Yellow River for YCO decreased by 21.56 × 10^8^ m^3^ in 2017, representing a decrease of 35.7%, and the average decrease over the 18 years was 1.2 × 10^8^ m^3^·a^−1^. The annual average groundwater depth of the entire YCO increased at a rate of 0.04 m, and the average groundwater depth increased by 0.74 m cumulatively over 18 years. Moreover, the groundwater depth in the Southern and Northern Irrigation Areas showed an upward trend, and the corresponding temporal and spatial distribution variations were quite different. The groundwater depth in the Southern and Northern Irrigation Areas increased at a rate of 0.03 m·a^−1^ and 0.05 m·a^−1^, respectively. After 2014, the groundwater depth increase in SIA slowed down, and the groundwater depth in the two areas increased by 0.57 m and 0.91 m, respectively, over 18 years. Compared with the SIA, the increase rate of groundwater depth in the NIA was nearly 1.7 times faster, making the groundwater depth in the NIA nearly close to that of the SIA in 2017. From the perspective of the irrigation period and non-irrigation period (Figure 3b), the groundwater depth increased at a rate of 0.06 m·a^−1^ and 0.02 m·a^−1^ respectively, which is three times faster than that in the non-irrigation period. The groundwater depth increased, with fluctuations, year by year in the irrigation period, but relatively leveled out in the non-irrigation period. By 2017, the groundwater depth in the two periods had increased to 2.74 m and 2.01 m respectively, an increase of 1.03 m and 0.45 m, respectively, over 18 years.

**Figure 4:**
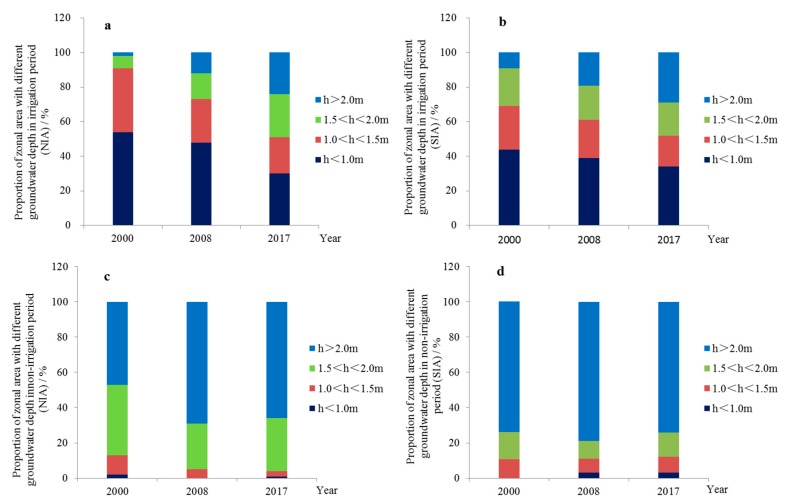
Proportion of zonal area with different groundwater depths: (**a**,**b**) Proportion of zonal area with different groundwater depths in the irrigation period, (**c**,**d**) proportion of zonal area with different groundwater depths in the non-irrigation period. NIA: Northern Irrigation Area; SIA: Southern Irrigation Area.

Based on the above differences in the spatial-temporal scale of groundwater depths, the proportion area of them with different depths was obtained. In the irrigation period (Figure 4a,b), the groundwater depth in most parts (91%) of NIA was less than 1.5 m in 2000, which was significantly reduced by 2017; the proportion area with a groundwater depth among 1.0 < h < 1.5 m, and h < 1.0 m decreased from 54% and 37% to 30% and 21%, respectively; and the proportion area with a groundwater depth greater than 1.5 m (1.5 < h < 2.0 m, h > 2.0 m) continued to increase significantly, from 2% and 7% to 25% and 24%, respectively. The groundwater depth in most parts (91%) of SIA was less than 2.0 m in 2000, which was significantly reduced by 2017, the proportion area with a groundwater depth of 1.5 < h < 2.0 m, 1.0 < h < 1.5 m, and h < 1.0 m decreased from 22%, 25%, and 44% to 19%, 18%, and 34%, respectively, and the proportion area with a groundwater depth greater than 2.0 m (h > 2.0 m) continued to increase, from 7% to 29%. It can be seen that in the NIA, as the proportion area with a groundwater depth less than 1.5 m gradually decreased and turned into those with a groundwater depth of 1.5 m or above, the proportion area with a groundwater depth greater than 1.5 m reached 49% in 2017, in which a groundwater depth greater than 2.0 m accounted for 24%. In SIA, as the proportion area with a groundwater depth less than 2.0 m gradually decreased and turned into those with a groundwater depth greater than 2.0 m or above, the proportion area with a groundwater depth greater than 1.5 m reached 48% in 2017, in which the area of zones with a groundwater depth greater than 2.0 m accounted for 29%.

In the non-irrigation period (Figure 4c,d), the groundwater depth in most parts of NIA was greater than 1.5 m (1.5 < h < 2.0 m, h > 2.0 m) in 2000, and in 2017, the area of the zones with a groundwater depth of 1.5 < h < 2.0 m, 1.0 < h < 1.5 m, and h < 1.0 m decreased from 40%, 11%, and 2% to 30%, 3% and 1%, respectively, making the area of zones with a groundwater depth greater than 2.0 m increase from 47% to 66%. Similarly, the groundwater depth in SIA was basically greater than 2.0 m (h > 2.0 m), and remained at 74% in 2017, while the area of zones with a groundwater depth between 1.0 m and 2.0 m (1.5 < h < 2.0 m, 1.0 < h < 1.5 m) decreased slightly, from 16% and 11% to 14% and 9%, respectively, and the zones with a groundwater depth less than 1.0 m (h < 1.0 m) accounted for 3%.

It is clear that, from 2000 to 2017, the zones with a groundwater depth greater than 1.5 m in NIA increased from 9% to 49% in the irrigation period, while in the SIA, it increased from 29% to 48%, and the proportion area of zones with different groundwater depth is basically the same; in the non-irrigation period, the zones with a groundwater depth greater than 1.5 m in NIA increased from 87% to 96%,where the zones with a groundwater depth greater than 2.0 m increased from 47% to 66%, while in SIA, it decreased from 90% to 88% and the zones with a groundwater depth greater than 2.0 m remained at 74%. Regardless of in the irrigation or non-irrigation period, the increase of proportion area in groundwater depth in NIA was greater than that in SIA, and the groundwater depths during the two periods were gradually the same as that in the SIA. 

#### 3.1.2. Variation Trend of Shallow Groundwater Storage after AWSAT 

**Figure 5:**
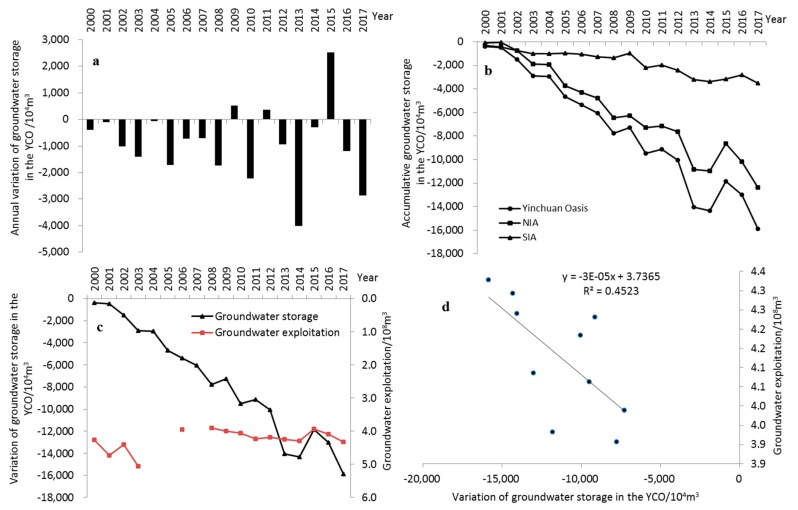
Groundwater storage variation and its relationship with groundwater exploitation: (**a**) Annual average groundwater storage variation, (**b**) accumulative groundwater storage variation, (**c**) variation of groundwater storage and groundwater exploitation, (**d**) correlation between groundwater storage variation and groundwater exploitation during 2008–2017. YCO: Yinchuan Oasis.

Groundwater storage in the YCO also changed profoundly after AWSTP. From 2000 to 2017, the variation of annual groundwater storage was basically negative, and groundwater has been in deficit for a long time, especially in 2013, when the maximum deficit reached 4000 × 10^4^ m^3^, i.e., the groundwater system was continuously in negative balance. At the same time, the accumulative groundwater storage decreased continuously, with an annual average decrease of 881.47 × 10^4^ m^3^ and a decrease of approximately 1.59 × 10^8^ m^3^ over 18 years. Among them, the Northern and southern irrigation areas saw a decrease of 1.24 × 10^8^ m^3^ and 0.35 × 10^8^ m^3^, respectively, and the decrease in NIA was higher than that in SIA (Figure 5a,b). 

#### 3.1.3. Analysis on the Driving Factors for Dynamic Changes of Shallow Groundwater after AWSTP 

**Figure 6:**
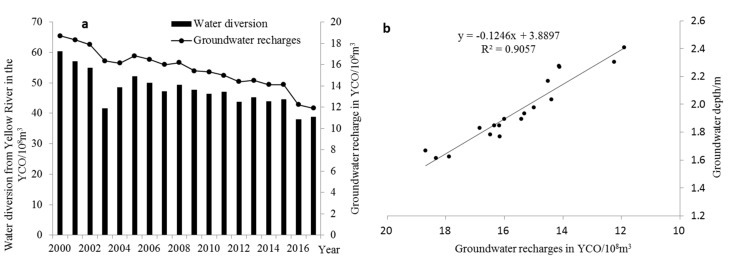
(**a**) Groundwater recharges variation with water diversion in the YCO, (**b**) relationship between groundwater recharge and groundwater depth.

After the implementation of the water allocation policies, due to the continuous decrease of the amount of water diverted from the Yellow River, the policy-related water shortage in the YCO has become prominent. In order to cope with agricultural water shortages and ameliorate the soil salinization caused by the heavy use of water diverted from the Yellow River, AWSTP were carried out in the YCO, including the optimization of water diversion and drainage systems, implementation of high-efficiency water-saving irrigation, adjustment of industrial structure, and other measures. First, in the early stage of AWSTP, the canal lining was mainly strengthened from the perspective of water diversion system optimization. Surprisingly, the average canal lining rate increased from 37.0%–45.0% in 1990s to 68.3% in 2015. Meanwhile, the high-efficiency water-saving irrigation was vigorously promoted in YCO, after which, the corresponding water-saving irrigation area increased from 2.43 × 10^4^ hm^2^ in 2000 to 8.5 × 10^4^ hm^2^ in 2017, representing an increase of 249.8%, and the water use efficiency increased by 81.7%, and remarkable results have been made in field water-saving. However, with the gradual decrease of field irrigation water and the continuous increase of canal lining, the canal seepage and the return water infiltration of field irrigation are dramatically decreasing, which will certainly have an important impact on groundwater recharge. Figure 6a shows that with the continuous decrease in the amount of water diverted from the Yellow River, the seepage recharge from the canal system and the field return water decreased continuously. The recharge decreased from 18.7 × 10^8^ m^3^ in 2000 to 11.9 × 10^8^ m^3^ in 2017, compared with 2000, an overall decrease of 36.3%, with an annual average decrease of 0.38 × 10^8^ m^3^. According to Pearson correlation analysis, Figure 6b shows a significant negative correlation between groundwater recharge and groundwater depth at the level of 0.05, with the correlation coefficient reaching −0.95. This shows that the decrease in groundwater recharge after the AWSTP is the root cause for the continuous increase in groundwater depth in the North–South direction (Figure 3) and the decrease in groundwater storage (Figure 5b) in the YCO during the irrigation period.

Second, after the AWSTP, the drainage system was also planned to gradually promote the ability of unblocking drainage, reducing underground water level, and controlling soil salinization. As such, on the one hand, the old ditches were dredged, several new drainage ditches were built by the government to subside water in fields and ditches, and the drainage ability was increased from 600.2 m^3^/s in the 1990s to 962.4 m^3^/s now. On the other hand, as one of prevalent measures, buried pipe drainage was combined with the ditch drainage in the YCO to discharge underground water. Until now, a total of 32 buried pipe drainage zones have been built in regions with poor drainage throughout the oasis, of which eight were distributed in SIA and 24 in NIA. The drainage by buried pipes mainly enters the first-level open ditches adjacent to the pipe network. The buried pipes are built with a depth of 1.2 m to 1.8 m, and the drainage period is from early June to late August, with a total drainage time of over 400 h, and the drainage quota for the whole growth period is 255–300 m^3^/hm^2^·h. According to the research of Wang et al. (2003), the average annual total drainage volume of ditches and berried pipe in NIA is 13.78 × 10^8^ m^3^, of which groundwater discharge accounts for about 27% [44], which is supposed to explain that under the same irrigation conditions, the buried pipe drainage accelerates the drainage in NIA, thus further lowering the groundwater level. This is one of the main reasons for higher lowering speed of the groundwater depth in NIA than that in SIA during the irrigation period and for great reductions in groundwater storage.

**Figure 7:**
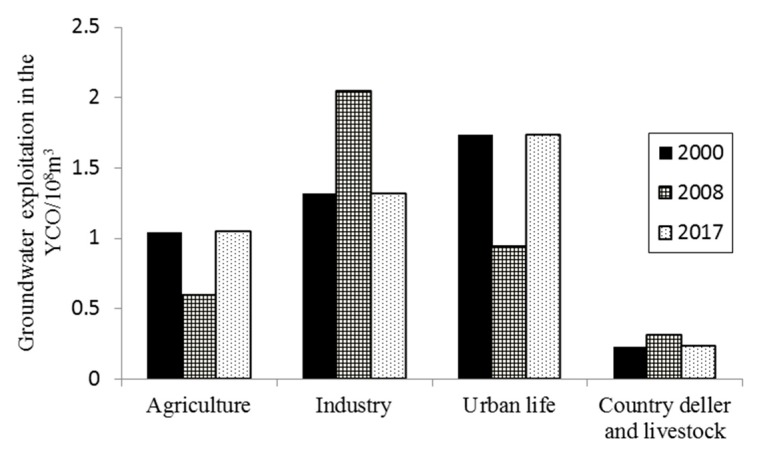
Groundwater exploitation of different industries in the YCO during 2000–2017.

Third, the implementation of the “well-canal combination” water resources optimal allocation mode in YCO is another important factor affecting the change in groundwater depth and storage. As it can be seen from Figure 5c, groundwater was free for exploitation without control before 2008, which was not clearly related to the decrease in groundwater storage. After 2008, with the further decrease in the amount of water diverted from the Yellow River and the further increase in the population, the exploitation of groundwater, especially deep groundwater, was gradually increased in YCO so as to meet the needs of agricultural irrigation and urban living (Figure 7), and the variation trend of groundwater storage was gradually consistent with that of groundwater exploitation. In 2013–2014, the groundwater exploitation reached a peak of 4.24–4.29 × 10^8^ m^3^, and coupled with the dry year, the cumulative decrease in groundwater storage reaching a maximum of 1.41–1.43 × 10^8^ m^3^ during 2013–2014. Until 2015, the total groundwater exploitation decreased, and the annual groundwater storage in 2015 turned to positive, and the reduction of accumulative groundwater storage also slightly slowed down. Groundwater dynamics in the non-irrigation period may have more to do with groundwater exploitation. The distribution of groundwater with different depths also shows that, compared with 2008, the groundwater depth in 2017 during the non-irrigation period hardly increased, and even a small amount of areas with a depth of less than 1.0 m was present in Southern and Northern Irrigation Areas (Figure 4c,d). This may be closely related to the gradual reduction of industrial groundwater extraction in YCO in recent years (Figure 7). According to the Pearson correlation analysis, a negative correlation between groundwater exploitation and groundwater storage from 2008 to 2017 is showed at the level of 0.05, with the correlation coefficient reaching −0.67 (Figure 5d). This indicates that groundwater exploitation in YCO, especially after 2008, has an important impact on the increase in groundwater depth and the decrease in groundwater storage.

Finally, the adjustment of agricultural and industrial structure and improvement of the utilization rate of irrigation water are another influence factor for the differential development of groundwater level and storage in Southern and Northern Irrigation Area during the irrigation period. Rice is the main food crop and water-consuming crop in YCO. Before AWSTP, rice was planted in a large area in NIA. A large amount of irrigation return water was infiltrated and became replenish groundwater, keeping the water table at a high level. Therefore, the planting area of rice in NIA was adjusted and the rice was replaced with wheat, corn and cash crops after the AWSTP with purpose of reducing irrigation water and lowering the groundwater level, the area of rice decreased by 14.5% (decreased from 6.48 × 10^4^ hm^2^ to 5.54 × 10^4^ hm^2^), saving about 1.01 × 10^8^ m^3^·a^−1^ of irrigation water. Utilization coefficient of the irrigation water thus increased from 0.32 to 0.52. At the same time, irrigation area of YCO was restrictively controlled at 32.6–33.1 × 10^4^ ha. The amount of irrigation return water infiltration was reduced, thus helping with the increase of groundwater depth in NIA to a certain extent and the reduction of groundwater storage.

Certainly, the spatial-temporal distribution of groundwater in YCO, especially during non-irrigation period, is also influenced by natural factors such as landform and topography, and climatic factors such as precipitation and evaporation. Due to the difference of topography and landforms, the terrain is steep in the South and flat in the North, and inclines from Southwest to Northeast. The ground slope reaches 1/1500–1/8000, and the groundwater flows from Southwest to Northeast. SIA is located in the groundwater runoff area in the South, with large groundwater hydraulic gradient, smooth flow of water and deep groundwater depth, while NIA is a stagnant area of groundwater flow, with flat terrain, small hydraulic gradient of groundwater, poor in drainage, susceptible to stagnant water and causing shallow groundwater depth. As a result, the spatial distribution pattern of groundwater depth from deep to shallow in the North-South direction in YCO has been formed. At the same time, YCO generally has less precipitation and strong evaporation. The shallower the groundwater depth is, the stronger the evaporation is. This is another factor that affects the groundwater depth more in NIA than SIA. However, with a groundwater depth gradually getting deeper in recent years, the groundwater evaporation capacity is also affected and weakened to some extent.

### 3.2. Spatial Distribution Variation of Groundwater Tds and Its Influence Factors

**Figure 8:**
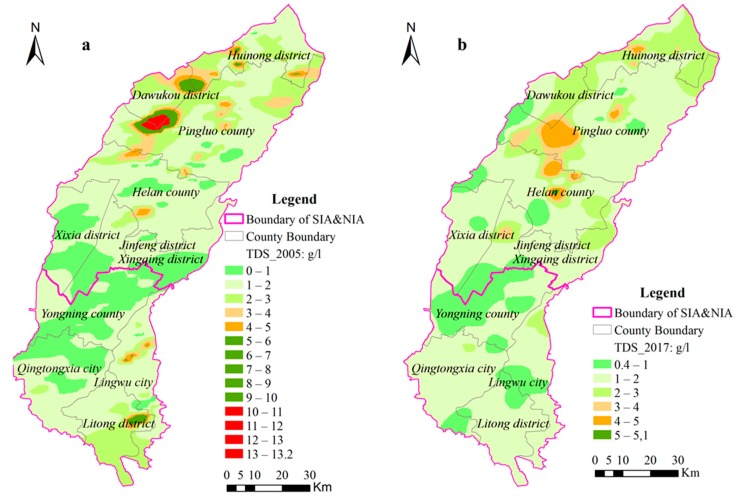
Spatial distribution of groundwater total dissolved solids (TDS) in the Yinchuan Plain: (**a**) TDS in 2005, (**b**) TDS in 2017.

**Figure 9:**
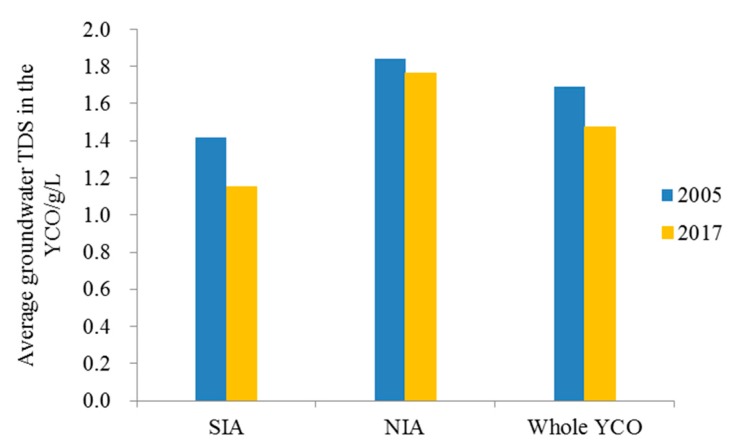
Average groundwater TDS in the YCO after the Agricultural Water-saving Transformation Project (AWSTP).

High groundwater salt is a typical groundwater quality problem in Yinchuan Oasis. As can be seen from Figure 8 and Figure 9, the average groundwater TDS of NIA and SIA has decreased from 1.84 g·L^−1^ and 1.41 g·L^−1^ to 1.77 g·L^−1^ and 1.15 g·L^−1^, respectively. Accordingly, the groundwater in most areas is brackish water (TDS is 1~3 g·L^−1^) and, average groundwater TDS in the SIA was a bit less than the NIA. Moreover, from 2005 to 2017, the spatial distribution difference of TDS in shallow groundwater has been generally decreasing and locally increasing. Among them, the TDS of Pingluo County and Dawukou District in Shizuishan City of NIA, and Lingwu City and Litong District in SIA tends to desalinize, especially in the vicinity of Shahu Lake in Pingluo County of NIA, where the saline water area and saltwater area (5–13.2 g·L^−1^) are gradually desalinated to brackish water areas with TDS of 3–5 g·L^−1^. Compared with 2005, the saline water areas with the TDS higher than 5 g·L^−1^ decreased by 16.6 km^2^ in 2017, the freshwater areas with the TDS lower than 1g·L^−1^ decreased by 334.4 km^2^, the brackish water areas with TDS of 1~3g·L^−1^ increased by 492 km^2^, and there is no saltwater areas with TDS exceeding 10g·L^−1^ (Table 3). It is worth noting that the TDS of groundwater has increased in local regions of Xixia District, Jinfeng District and Xingqing District, Yongning County and Helan County in the middle of NIA and Huinong District at the Northern end.

In Yinchuan Oasis, due to the regional hydrogeological conditions, a considerable amount of the soluble salt in the region comes from the precipitation leaching rock strata into the shallow groundwater, and taking the soluble salt dissolved in the aquifer into account, the background value of groundwater TDS is relatively high. At the same time, the soluble salt in phreatic water moves from Southwest to Northeast along with groundwater runoff, so the NIA becomes the main place for soluble salt accumulation due to stagnant runoff and poor groundwater discharge, thus forming the different spatial distribution pattern of TDS in SIA and NIA. The desalinization of groundwater TDS that appeared after the AWSTP is, on the one hand, due to the South–North differentiation of groundwater depth and decreasing water diversion in YCO. In Yinchuan Oasis, especially in the SIA, when water diversion decreased, the shallow groundwater recharge from canal water leakage and irrigation water returned flow infiltration was decreased, and the soluble salt infiltrated with the water into shallow groundwater was also reduced. On the other hand, some soluble salt is drained out of the oasis along with groundwater through ditches drainage, berried pipe drainage, and groundwater discharge, etc., and the amount of drained salt is always enormous, thus slightly reducing the overall salinization of shallow groundwater. Through comparison between Figure 8 and Table 3, it is found that most of the areas with increased groundwater TDS in NIA are converted from freshwater areas with TDS lower than 1 g·L^−1^ to brackish water areas with TDS of 1–3 g·L^−1^. The reason for this is that the Xixia, Jinfeng and Xingqing Districts, Yongning County, and Helan County in the middle of NIA are located in urban areas, and with the increase in the population, a large amount of groundwater has been exploited in recent years, which has resulted in the deepening of groundwater depth and the evaporation threshold exceeding 3 m; in some places, groundwater depression cones even occurred, which prevents the soluble salt in the groundwater from being effectively evaporated and entering the soil layer, and thus accumulating in the groundwater. At the same time, some soluble salt in the surrounding areas and recharge water migrates with groundwater flow into these areas with relatively deep groundwater depth, thus slightly increasing the groundwater TDS. In addition, the TDS in groundwater is constantly changing with the movement of groundwater flow. When irrigation and drainage conditions are changed, the concentration, path, and direction of soluble salt migration will also change, thus affecting the spatial distribution of groundwater TDS.

## 4. Conclusions

The research above shows that the groundwater depth in Yinchuan Oasis, no matter in Southern or NIA, is deepening and the accumulative groundwater storage is decreasing after water-saving transformation. At the same time, the groundwater TDS is slightly desalinized in most areas, and it is mainly brackish water with TDS of 1–3 g·L^−1^. The areas of saline water Ⅰ with TDS of 3–5 g·L^−1^ and saline water Ⅱ with TDS of 5–10 g·L^−1^ are reducing, while brackish water appears in local areas of Xixia, Jinfeng and Xingqing Districts, Yongning, and Helan County in the mid-NIA. The spatial and temporal distribution of groundwater and salinity is affected to a certain extent by natural factors such as topography, geology, and climate, while the circulation pattern of groundwater and salinity and its differentiation are closely related to agricultural water-saving activities caused by the Yellow River water allocation policies. The agricultural water-saving measures (water diversion and drainage, groundwater exploitation, industrial structure adjustment), especially the reduction of water diverted from the Yellow River, not only change the conditions for shallow groundwater recharge and drainage, but also fundamentally change the spatial distribution and cyclic redistribution process of groundwater and salinity. This change has played a positive feedback role, for example, the water use efficiency during this period is improved and the groundwater salinity is generally desalinated. In the long run, the continuous decrease of groundwater recharge and storage and the increase of groundwater depth may have unexpected negative influences on regional groundwater and salt circulation and ecological environment health. Although the effect of agricultural water-saving measures on small-scale water-saving in the field is worthy of recognition, its influence on the groundwater and salt circulation process in large-scale areas shall also be fully taken into account. Therefore, for Yinchuan Oasis, the variation relationship among agricultural water use, ecological water use, and groundwater environment changes shall be coordinated, water diversion and drainage measures shall be optimized based on the actual water demand and allocation, and the groundwater exploitation and industrial structure shall be rationally allocated, ensuring that the groundwater and salt circulation in Yinchuan Oasis are in a good, healthy and sustainable development state. 

## Figures and Tables

**Table 1:** Key parameters and their variability for the semi-variogram fitting of TDS.

Year	Model	Range (m)	Lag Size	Nugget (C_0_)	Partial Sill (C)	Spatial Correlation (C0/(C0 + C)) (%)
2005	Spherical	19,920	1660	0.2	0.09	68.97
2017	Spherical	13,091	1873	0.17	0.11	60.71

**Table 2:** Error evaluation of semi-variogram of TDS.

TDS	Mean Error (ME)	Standardized Mean Error (SME)	Root-Mean-Square Error (RMSE)	Average Standard Error (ASE)	Root-Mean-Square Standardized (RMSSE)
2005	−0.06	−0.09	0.525	0.49	1.34
2017	−0.01	−0.01	0.46	0.37	0.91

**Table 3:** Classification area and proportion of groundwater TDS in the Yinchuan Plain.

Year	District	Classification	Grade of Total Dissolved Solids				
			<1 g·L^−1^	1–3 g·L^−1^	3–5 g·L^−1^	5–10 g·L^−1^	>10 g·L^−1^
2005	NIA	Area/km^2^	680.4	2940.5	324.8	77.0	34.9
		Proportion/%	16.9	73.1	8.1	1.9	0.9
	SIA	Area/km^2^	690.7	1503.7	30.1	24.7	0.0
		Proportion/%	30.7	66.9	1.3	1.1	0.0
2017	NIA	Area/km^2^	451.9	3272.6	316.5	16.6	0.0
		Proportion/%	11.1	80.7	7.8	0.4	0.0
	SIA	Area/km^2^	584.7	1663.7	0.8	0.0	0.0
		Proportion/%	26.0	74.0	0.0	0.0	0.0
